# Signature transcriptome analysis of stage specific atherosclerotic plaques of patients

**DOI:** 10.1186/s12920-022-01250-8

**Published:** 2022-04-29

**Authors:** Sonia Verma, Abhay Kumar, Rajiv Narang, Akshya K. Bisoi, Dipendra K. Mitra

**Affiliations:** 1 Division of Neuroscience and Ageing Biology, CSIR-Central Drug Research Institute, Lucknow, Uttar Pradesh India; 2 Department of Microbiology, Indira Gandhi Institute of Medical Sciences, Patna, India; 3 Department of Cardiology, All India Institute of Medical Sciences, New Delhi, India; 4 Department of Cardiothoracic and Vascular Surgery, Cardio, and Neurosciences Center, AIIMS, New Delhi, India; 5 Department of Transplant Immunology and Immunogenetics, All India Institute of Medical Sciences (AIIMS), Room No-75, New Delhi, 110029 India

**Keywords:** mRNA, miRNA, Atherosclerosis, Biomarker, Plaque stage

## Abstract

**Background:**

Inflammation plays an important role in all the stages of atherosclerotic plaque development. The current study aimed at assessing the altered expression of genes functioning in inflammation within the early stage (ES) and advanced stage (AS) atherosclerotic plaques obtained from patients undergoing coronary artery bypass grafting (CABG) surgery and identifying biomarker panel/s that may detect the status of plaque stages using peripheral blood samples.

**Methods:**

A section of ES and AS plaques and normal left internal mammary arteries (LIMA) were obtained from 8 patients undergoing the CABG surgery. Total RNA isolated was analyzed for mRNA and miRNA expression profile by Affymetrix arrays. A significant number of mRNAs was found to be differentially expressed in ES and AS plaque tissues relative to LIMA. The pathway analysis of differentially expressed mRNAs in the two plaque stages was also performed using DAVID Bioinformatics Database.

**Results:**

The mRNAs were found to be involved in critical inflammatory processes such as the toll-like receptor signaling pathway and cytokine-cytokine receptor interaction. Few miRNAs targeting these mRNAs were also altered in the two plaque conditions. QRT-PCR results showed a similar expression pattern of a few of the mRNAs and miRNAs in peripheral blood of the same patients relative to healthy controls.

**Conclusion:**

Changes in mRNA and miRNA expression associated with various inflammatory processes occur in different atherosclerotic stage plaques as well as peripheral blood. Detection of such variations in patients’ blood can be used as a possible prognostic tool to detect and/or predict the risk and stage of atherosclerosis.

**Supplementary Information:**

The online version contains supplementary material available at 10.1186/s12920-022-01250-8.

## Background

Atherosclerotic vascular plaques are the major pathological basis for cardiovascular diseases (CVD) including ischemic heart disease (IHD), stroke, peripheral vascular disease, and renovascular hypertension. Inflammation is involved in the majority of atherosclerosis stages. The initial phase of atherosclerosis is mediated by the subendothelial accumulation of lipid molecules, including low-density lipoproteins (LDL), which later undergo oxidation by free radicals and oxidative enzymes [1, 2]. Accumulation of damaged mitochondrial DNA in circulation and immune cells is also one of the factors promoting inflammation during atherosclerosis [3, 4]. The expression of various chemokines and different adhesion molecules is stimulated with further recruitment, adhesion, and activation of circulating monocytes, other innate and adaptive immune cells as well as activated thrombocytes to the endothelium. VSMCs also migrate to the lesion site, transdifferentiate to plaque macrophages, express pro-inflammatory cytokines, and perform phagocytosis [5]. These cells along with circulating monocytes accumulate atherogenic modified LDL particles, such as oxidized LDL or desialylated LDL, and form foam cells, a characteristic feature of atherosclerotic plaques [1, 2].

At later stages of the development, the plaque is separated from the vessel lumen by a fibrous cap. At advanced stages, atherosclerosis is associated with prominent vascular wall thickening and calcification [6]. Both macrophages and VSMCs can release calcifying extracellular vesicles (EVs) [7]. The earliest calcifications also called microcalcifications (size < 50 μm), majorly originate from the lipid pool and early necrotic core, but can also be formed in the fibrous cap. Microcalcifications further exaggerate the inflammatory response [8]. The resulting pro-inflammatory response amplifies several processes related to microcalcification, triggering a battery of subsequent complications including rupture of the thrombotic plaque and/or blockade of vascular lumen leading to ischemic pathologies [9].

As the vascular plaque evolves, it converts from an early, lipid-rich atheroma to an advanced, fibro-calcified lesion [9]. Lipid-rich plaques with a thin fibrous cap are prone to rupture, precipitating in thrombus formation, leading to vascular occlusion causing acute ischemia and infarction of tissue. Methods to determine the nature of plaque are generally invasive and cumbersome. However, despite major advances in treatment, significant numbers of coronary artery disease (CAD) patients develop acute coronary syndromes and even have sudden cardiac death. Early detection of vulnerable plaque can identify patients at high risk for such events. Therefore it has become critical to develop improved molecular signature profiles for screening the plaque formation, preferably with stage specific signatures. Development of improved prognostics biomarker(s) and thus, novel therapeutic strategies for CVD, necessitates better understanding and identification of various key molecules and genetic pathways uniquely involved in various stages of atherosclerotic plaque formation.

Attempts toward the analysis of the gene expression profiles in atherosclerotic plaques are reported [10–14]. For comparative study, these reports used samples from different individuals, which may confound the results due to inter-individual genetic variation, tissue-specific differences in transcriptomes, and differences in systemic parameters. In CAD patients, distinct regions with ES or AS plaques may coexist within a single artery, hinting toward pathogenic attribute(s) leading to their sequential and/or differential evolution [15, 16]. Previous studies are mostly based on using autopsied specimens which may not truly represent the in vivo changes of the plaque. Therefore, to have better insights into the molecular profile of gene expression in the various stages of atherosclerotic plaques, we performed a microarray analysis of early stage (ES) and advanced stage (AS) plaques as well as healthy Left Internal Mammary Artery (LIMA) tissues obtained surgically from the same live individuals undergoing coronary artery bypass grafting (CABG) surgery.

We identified several inflammatory processes and pro-inflammatory cytokines associated with these plaques, evidencing a sustained inflammation and cytokine storm in the patients. Also, the miRNAs targeting these genes were found to be differentially expressed in the respective plaque stage. The expression levels of some of these genes were found to be following the same trend in patients’ blood as observed in the plaques. Conclusively, our data provides for an early and faster prognostic tool for detecting and predicting the risk and stage of atherosclerosis.

## Methods

### Tissue collection

Patients’ samples were obtained from 8 individuals (males-5, females-3), aged 55–80 years, undergoing CABG surgery at the apex tertiary referral center, All India Institute of Medical Sciences (AIIMS), New Delhi, India. Plaque tissues excised at the time of surgery as well as a 5–8 mm segment of LIMA were taken for analysis. The plaque tissue was divided into two parts depending upon the extent of calcification and lipid content. We used ES/lipid-rich plaque and AS/fibro-calcified plaque for further study. All these tissue fractions and LIMA segments were frozen immediately in QIAzol and stored at − 80°C before RNA extraction. The study has been approved by the Institutional Ethics Committee, All India Institute of Medical Sciences, New Delhi, India, and all patients signed informed written consent.

### RNA isolation

RNA was extracted from tissues using Qiagen miRNeasy Mini Kit. The tissues were placed in a 2 ml sterile Eppendorf tube containing RLT lysis buffer (Qiagen®). Samples were then homogenized individually on ice with a mechanical tissue homogenizer turned to the maximum position (3000 rpm) in short pulses of 15 seconds each for 10 cycles. The homogenates were separated into aqueous and organic phases by centrifugation after adding chloroform. The upper, aqueous phases containing RNA were extracted. After adding ethanol, the samples were applied to the RNeasy Mini spin column for total RNA binding to the membrane. Following washes, total RNAs were eluted in RNase-free water. The concentrations of the RNA were determined using NanoDrop 2000 (Thermo Scientific, USA). RNA from peripheral blood was also isolated using the same kit.

### Microarray analysis

For microarray analysis, RNA from tissues with the similar condition of 4 patients was pooled to make one batch for sequencing. RNA pooling as a strategy before performing microarray experiments has been widely reported to optimize both the cost of data generation as well as the statistical power for differential gene expression analysis [17–19]. The biggest advantage of pooling occurs when the biological variability is large [17]. Two batches, each having 3 different samples, representing ES, AS and LIMA were sent for sequencing to Imperialls Life Sciences (P) Limited, India. In brief, for mRNA, cDNA synthesis, amplification, and gene expression profiling were done with the GeneChip WT PLUS Reagent Kit (Affymetrix) and labeled with the Genechip hybridization wash and stain kit (Affymetrix). Samples were hybridized with Gene Chip Human Transcriptome Array (HTA) 2.0 (Affymetrix) following the manufacturer’s protocol. For miRNA, total RNA was labeled with Biotin using the AffymetrixFlashTag™ Biotin HSR kit following the manufacturer’s protocol. Labeled extracts were hybridized to the GeneChip miRNA 4.0 Arrays overnight and subsequently processed using GeneChip Hybridization Wash and Stain kit (Affymetrix) according to the manufacturer’s instructions.

### Differential gene expression analysis

Transcriptome Analysis Console [TAC] 3.1 software [Affymetrix, CA, USA] provided by the Affymetrix Corporation was used to analyse significantly differentially expressed genes [fold change > 2 or fold change < − 2] using a default algorithm one-way between-subject ANOVA [unpaired] and a filter criteria ANOVA p-value [Condition pair] < 0.05.

### Functional enrichment of genes

The online tool, DAVID Bioinformatics Resources analyses was used to conduct the Kyoto Encyclopedia of Genes and Genomes (KEGG) pathway enrichment analysis of statistically significant, differentially-expressed mRNAs [20–22]. The *p-*value< 0.05 was set as the cut-off for selecting significantly over-represented pathways.

### miRNAs targeting differentially expressed mRNAs

Differentially expressed mRNAs were subjected to the online database miRWalk (http://mirwalk.uni-hd.de/), where correlations between miRNAs and their target genes have been experimentally confirmed. In the database, three algorithms including targetScan, miRDB, and miRTarBase are used to predict miRNAs targeting the gene of interest. Any miRNA predicted for a given mRNA by all the three algorithms was considered to be targeting that gene.

### MiRNA-target genes correlation

The reverse correlation between mRNAs and the miRNAs targeting these genes was screened by overlapping the predicted miRNAs of up-regulated mRNAs with the identified down-regulated miRNAs and vice-versa for the respective plaque tissue relative to LIMA.

### QRT-PCR

1μg of RNA isolated from peripheral blood was reverse transcribed and used for miRNA quantification using Mir-X^TM^ miRNA First-Strand Synthesis and TB Green qRT-PCR kit (Takara Bio Inc.). U6 was used as an internal control. For mRNA quantification, about 1μg of RNA isolated from peripheral blood was converted to cDNA with the cDNA Reverse Transcription Kit (Applied Biosystems, USA). Quantitative RT-PCR (qRT-PCR) was carried out on RealPlex PCR system (Eppendorf, USA) with SYBR Premix Ex Taq^TM^ (TaKaRa Bio Inc.). GAPDH was used as the internal control. Statistical analysis was performed using GraphPad 7.0. The primers used are enlisted in Additional file 1.

## Results

### Differential gene expression profiling in ES and AS plaque

**Fig. 1:**
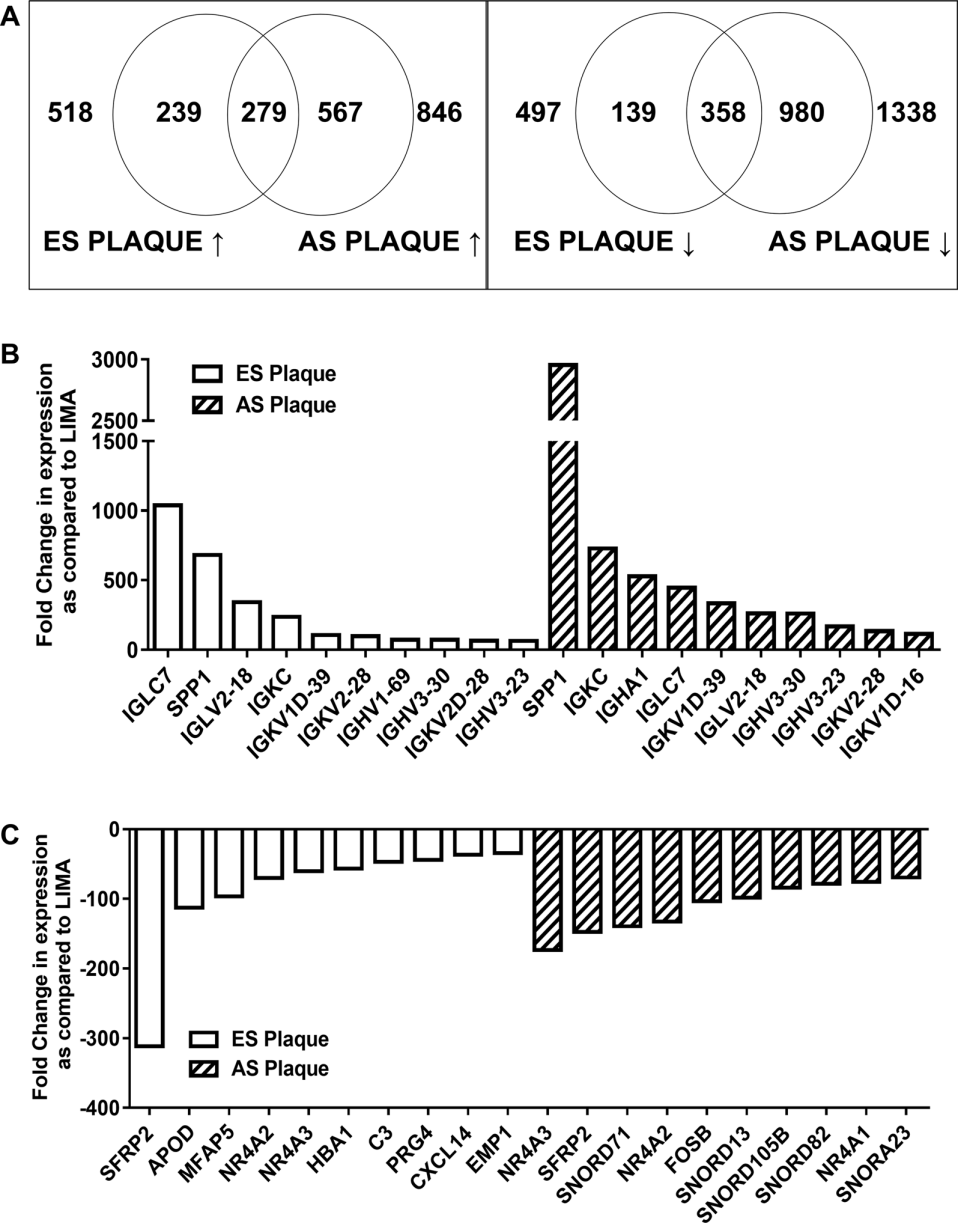
Microarray analysis reveals alterations in gene expression associated with atherosclerotic plaques. **a** Venn diagram of differentially expressed genes in early (ES) and advanced stage (AS) atherosclerotic plaque as compared to Left Internal Mammary Artery (LIMA) (↑—up-regulated; ↓—down-regulated). **b** Bar graph of top 10 mRNAs significantly up-regulated as compared to LIMA in microarray analysis. **c** Bar graph of top 10 mRNAs significantly down-regulated as compared to LIMA in microarray analysis. n = 2 (each with sample pooled from 4 patients); *p* < 0.05

To investigate the variation in the expression of genes in human ES and AS atherosclerotic plaques, microarray sequencing and analysis of total RNA isolated from both the plaque tissues as well as LIMA was carried out. For ES plaques, 518 genes were significantly up-regulated and 497 genes were significantly down-regulated relative to LIMA (Fig. 1a). In AS plaque samples, we found more genes to be differentially expressed, i.e. 846 up-regulated genes while 1338 down-regulated genes as compared to LIMA (Fig. 1a). Interestingly, in both the plaques 279 and 358 common genes were up and down-regulated, respectively (Fig. 1a). The list of the top 50 differentially expressed mRNAs is provided in Additional file 2. Some of these genes such asSPP1, TGFB, and AKT3 have been associated with atherosclerosis in previous studies on mice and human samples, thus supporting our findings [23–28]. The topmost 10 up-regulated and down-regulated mRNAs are presented in Fig. 1b and c. In summary, the microarray data indicates that atherosclerotic plaques are associated with alterations in gene expression which may further differ among various plaque stages.

### ES Plaques are associated with the up-regulation of inflammatory mediators

**Fig. 2:**
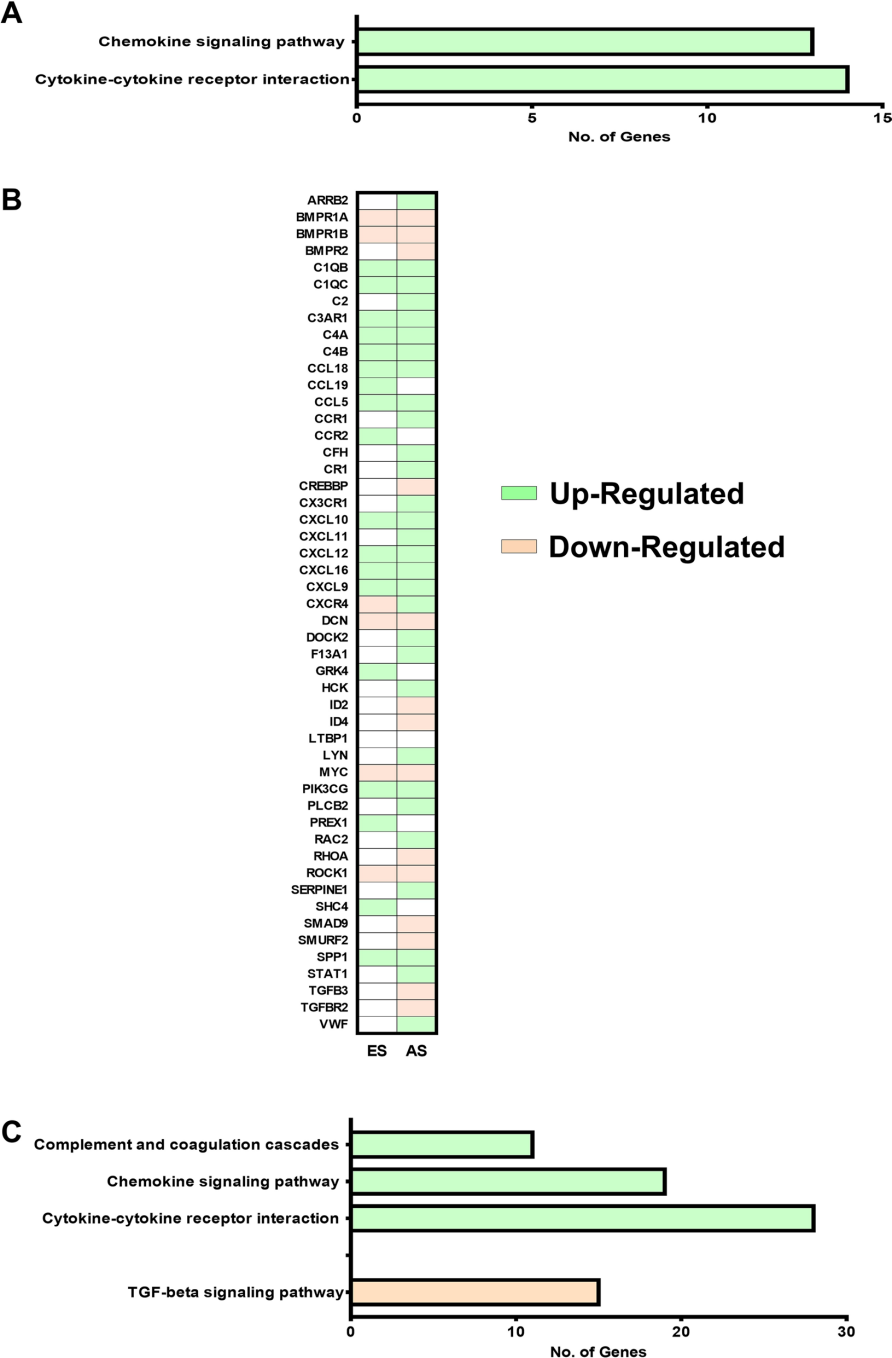
The differentially expressed genes in the plaques are associated with inflammation. **a** Bar graph showing significantly-enriched inflammation associated KEGG pathways of differentially expressed genes in early stage (ES) atherosclerotic plaques. The KEGG pathway analysis was done using an online database, DAVID. **b** List of differentially expressed genes functioning in inflammation pathways mentioned in A and C. **c** Bar graph showing significantly-enriched inflammation associated KEGG pathways of differentially expressed genes in advanced stage (AS) atherosclerotic plaques. n = 2 (each with sample pooled from 4 patients); *p* < 0.05

Inflammation plays an important role in atherosclerosis. Elevated levels of inflammatory cytokines and chemokines have been observed in various studies on affected cardiac tissues [10–14]. In the current study, we delineated the genes involved in the inflammation of ES and AS plaques by KEGG pathway analysis of differentially expressed genes using DAVID. In ES plaques, 19up-regulated genes were related to the chemokine signaling pathway and cytokine-cytokine receptor interaction (Fig. 2a, Additional file 3). Interestingly, most of these pro-inflammatory genes like CCL5, CXCL10, CXCL9, CTSK, and CD14 have been previously reported to be elevated in the serum of patients with coronary artery diseases [13, 14, 29–32]. The list of differentially expressed genes functioning in inflammation pathways is shown in Fig. 2b.

**Fig. 3:**
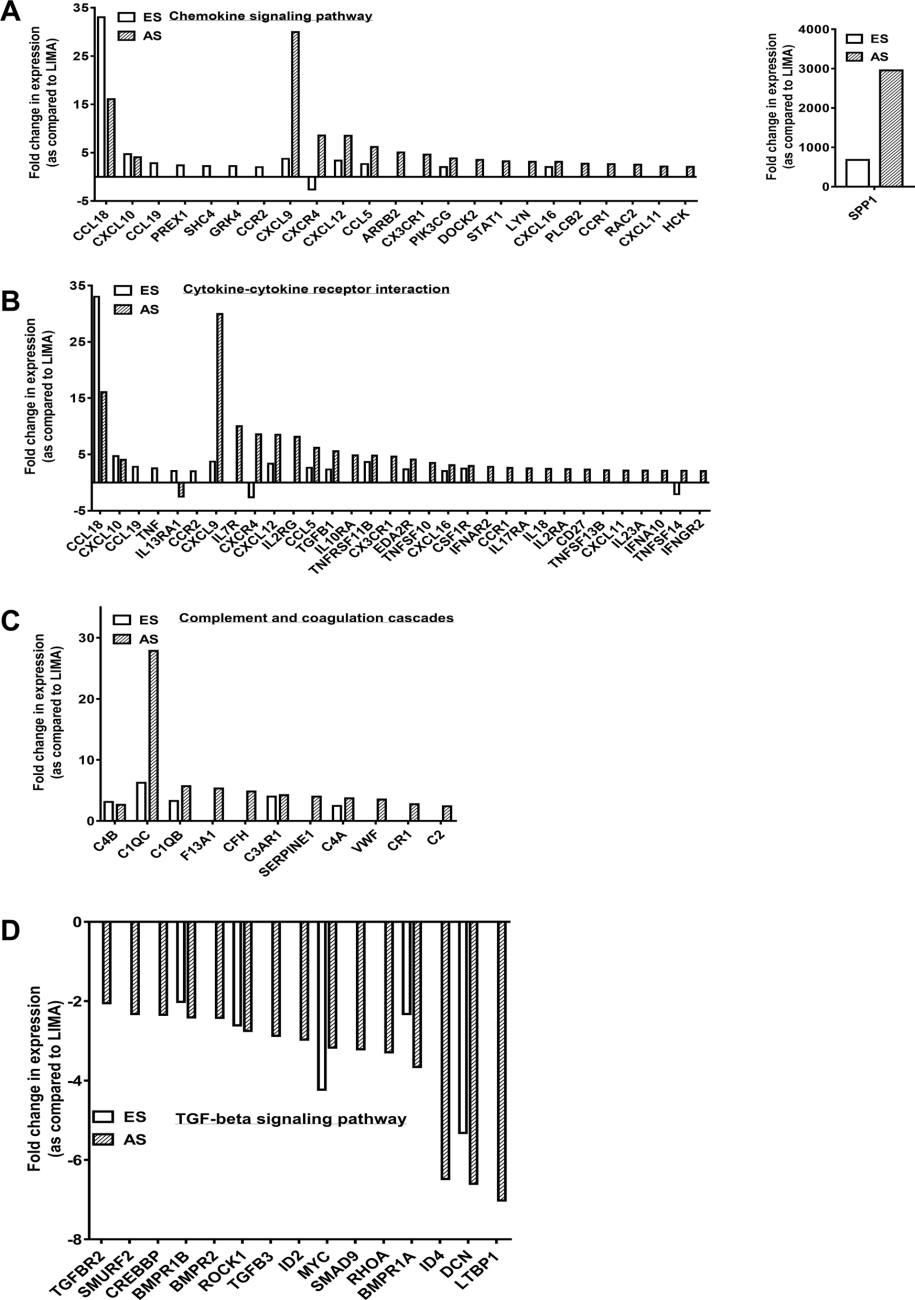
Expression of various inflammatory pathways-associated genes is altered in atherosclerotic plaques. Graphs showing microarray-analysis-based fold change in expression of genes in early stage (ES) and advanced stage (AS) atherosclerotic plaques as compared to (Left Internal Mammary Artery) LIMA. Using an online database DAVID the genes were found to be involved in chemokine signaling pathway (**a**), Cytokine-cytokine receptor interaction (**b**), Complement and coagulation cascades (**c**), and TGF-beta signaling pathway (**d**). n = 2 (each with sample pooled from 4 patients); *p* < 0.05

Few of these 19 genes were also found to be up-regulated in AS plaques while the expression of remaining genes was found only in ES plaque; for example, PREX1, SHC4, and CCR2 (Fig. 3a and b). These ES plaque-specific genes function in regulating the signaling pathways for chemotaxis and migration of various immune cells. Since these genes show plaque-specific up-regulation, they can be used as biomarkers to indicate the presence of ES plaque in patients.

### AS plaques are also associated with the up-regulation of inflammatory mediators

Upon pathway analysis for up-regulated genes in AS plaques, 48 of these genes were found to be involved in complement and coagulation cascades along with chemokine signaling pathway and cytokine-cytokine receptor interaction (Fig. 2c, b, Additional file 3). Among the genes functioning in the chemokine signaling pathway and cytokine-cytokine receptor interaction, 23 were found to be up-regulated specifically in AS plaque (Fig. 3a and b). We also observed that the few genes functioning in the chemokine signaling pathway and cytokine-cytokine receptor interaction, whose expression was enhanced in both ES and AS plaque, showed higher fold change in the later stage (Fig. 3a and b). Among these genes are SPP1, CXCL9, CXCL12, and CCL5.

The up-regulated genes involved in Complement and Coagulation Cascade include C1QB, C1QC, CFH, and C2 (Fig. 2b and c, Additional file 3). These genes function in the classical complement pathway which is activated not only during bacterial infections but also in the absence of proper immune regulatory mechanisms. Therefore, in AS plaque, the activation of this system might be involved in the clearance of damaged cells. In summary, the AS plaque is not only associated with a greater number of highly expressed genes but also shows a higher fold change of commonly up-regulated genes as compared to ES plaques. Examining the expression levels of such genes in peripheral blood of patients would suggest the prevalence of plaque stage and support the clinicians in further management of the disease.

### AS plaques are associated with down-regulation of anti-inflammatory mediators

The inflammatory response must be actively terminated when no longer needed to prevent unnecessary "bystander" damage to tissues. Failure to do so results in chronic inflammation, and cellular destruction. We found a similar observation in AS plaques; 15 down-regulated genes were involved in the anti-inflammatory-TGF-beta signaling pathway (Fig. 2c, Additional file 3). The loss of expression of some of these genes like BMPRII (receptor of the TGF-beta) and DCN (cellular matrix proteoglycan that binds to TGF-beta) has been reported in human coronary arteries with advanced atherosclerotic lesions further supporting our observations and also suggesting an unchecked hyper-inflammatory condition in AS plaques [33]. Few of these genes like BMPR1B, ROCK1, and DCN were also found to be down-regulated in ES plaque as well and their fold change difference was similar to that found in AS plaque (Fig. 3d).

### Distinct miRNA profiles in ES and AS plaque

**Fig. 4:**
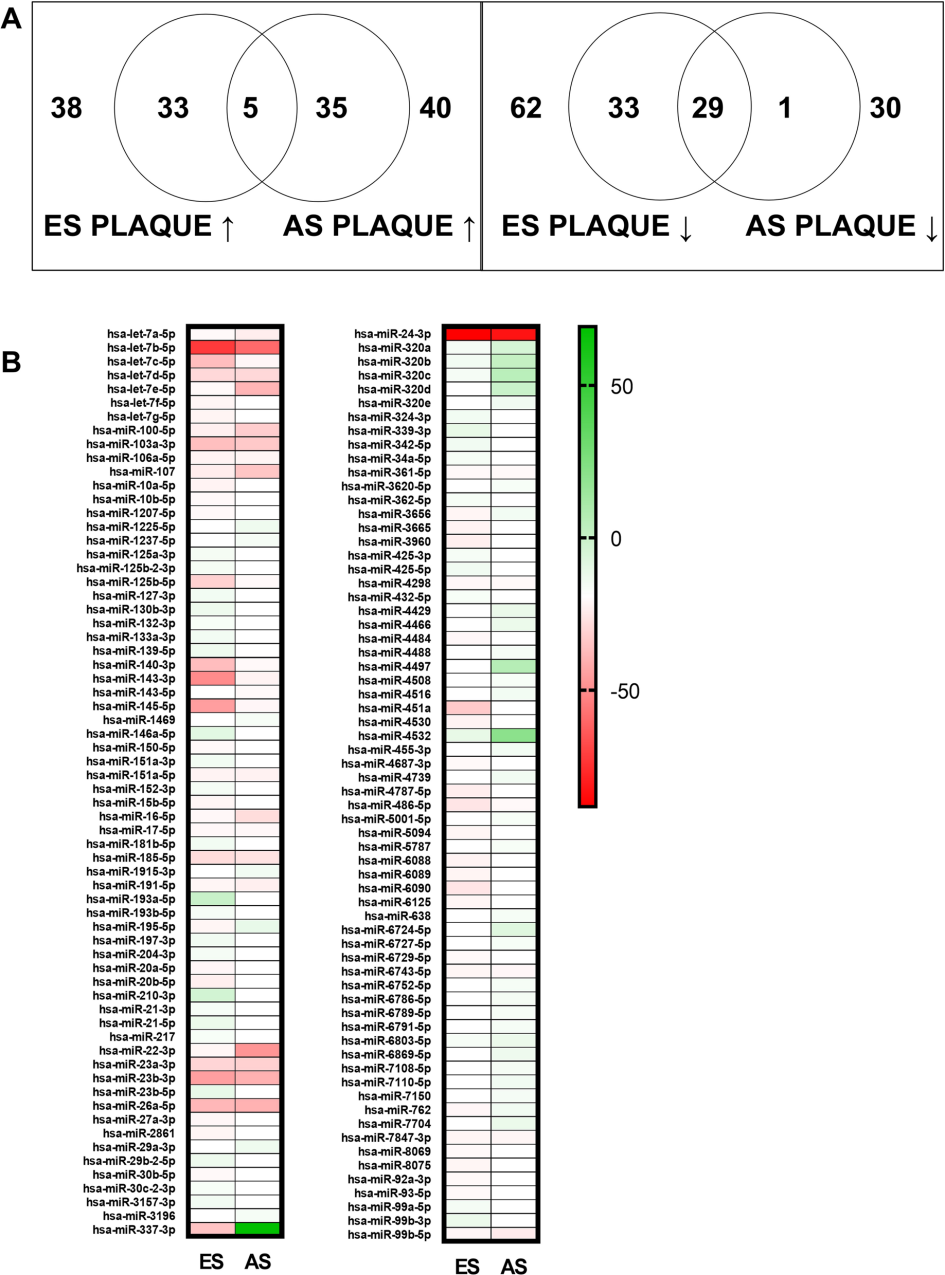
Atherosclerotic plaques are associated with alterations in miRNA expression. **a** Venn diagram of differentially expressed miRNAs as found in microarray analysis in early (ES) and advanced stage (AS) atherosclerotic plaques as compared to Left Internal Mammary Artery (LIMA)**. b** Heat-map of miRNAs differentially expressed in the two plaques. n = 2 (each with sample pooled from 4 patients)

The expression levels of 132 miRNAs differed in the two plaque tissues relative to LIMA. However, the variation in the expression of these miRNAs was insignificant in our data. This might be due to the small sample size. Of these miRNAs, 38 were up-regulated and 62 were down-regulated in the ES plaques when compared to LIMA (Fig. 4a and b). In AS plaques, 40 miRNAs were up-regulated and 30 miRNAs were down-regulated as compared to LIMA (Fig. 4a and b). Also, 5 miRNAs were up-regulated and 29 miRNAs were down-regulated in both the plaques (Fig. 4a and b). The full list of differentially expressed miRNAs is provided in Additional file 4. Recently, the down-regulation of miR-143 has been observed in advanced coronary atherosclerotic plaques [34]. Mir-143 is thought to be involved in cardiac morphogenesis and cancer [35]. In human aortic aneurysms, the expression of mir-143 has also been found to be significantly decreased when compared to controls [36]. In our study, we report down-regulation of this miRNA not only in AS plaque but also in ES plaque. Interestingly the fold change was much lower in ES plaque (− 40) than AS plaque (− 4), suggesting its possible role in altering vascular smooth muscle cells morphology early during atherosclerosis.

The two miRNAs that were up-regulated in ES showed down-regulation in AS plaque. Of these, miR-22 is reduced in human arteries from arteriosclerosis obliterans [37]. The down-regulation of miR-22 increases the expression of pro-inflammatory cytokines and therefore suggests the creation of an unfavorable pro-inflammatory situation that promotes plaque formation [38]. We also found 4 miRNAs, down-regulated in ES plaque, to be up-regulated in AS plaque. In summary, the miRNA expression analysis shows an association of atherosclerotic plaques with variable expression of different miRNAs, and the presence of a similar trend of expression of these miRNAs in ES and As plaques can be used as a potential biomarker.

### Negative correlation between miRNAs and mRNAs profile in atherosclerotic plaque

The small non-coding RNAs-miRNAs are known to regulate the expression of various genes by inhibiting their translation. To understand the role of miRNAs in atherosclerosis-associated inflammation, using the miRWalk database we first predicted the miRNAs for the inflammatory mediators that were up-regulated in ES and AS plaque as compared to LIMA. Next, these miRNAs were then overlapped with the miRNAs that were found to be down-regulated in the microarray analysis of respective tissue. A Similar methodology was employed for down-regulated inflammatory mediators and the miRNAs targeting them. In this manner, miRNA-target gene interaction pairs of reverse association in the two plaque tissues were obtained. We found 3 such interacting pairs with up-regulated mRNAs and down-regulated miRNAs (Table 1). 2 of these pairs included miRNAs (hsa-miR-125b-5p and hsa-miR-23a-3p) down-regulated in AS plaque. Interestingly, the target gene of hsa-miR-125b-5p and hsa-miR-23a-3p- TNFAIP3 was also found to be up-regulated in the AS plaque. The remaining interaction pair included miRNA, hsa-miR-22-3p, and its target CSF1R. In both the plaques, this miRNA was down-regulated while its target gene was up-regulated (Table 1). The opposite trend of expression of the above-mentioned miRNAs and mRNAs in patients’ blood can further support the diagnosis of plaque stage.

**Table 1:** miRNA-target gene interaction pairs of reverse association in the two plaque tissues

	miRNA	Fold change vs LIMA	Target mRNA	Fold change vs LIMA
ADVANCED STAGE	hsa-miR-125b-5p	− 2.26	TNFAIP3	4.88
	hsa-miR-23a-3p	− 15.88	TNFAIP3	4.88
EARLY STAGE	hsa-miR-22-3p	− 3.32	CSF1R	2.49
ADVANCED STAGE	hsa-miR-22-3p	− 36.7	CSF1R	2.95

### Distinct mRNA/miRNA profiles are detected in patients’ blood

**Fig. 5:**
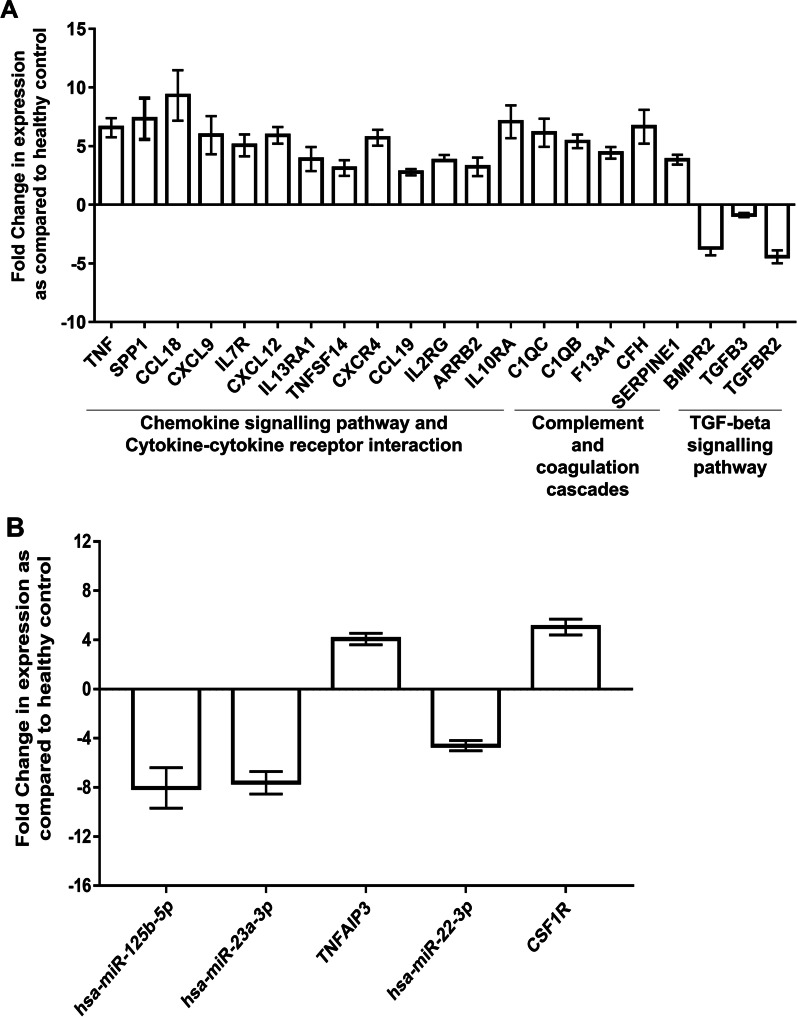
The Expression of mRNA/miRNA is also altered in the peripheral blood of patients. **a** QRT-PCR based quantification of mRNAs of a proposed biomarker panel in peripheral blood of patients and comparison with healthy control. **b** QRT-PCR based quantification of identified miRNA-mRNA interaction pairs in peripheral blood of patients and comparison with healthy control. GAPDH mRNA was used for normalization. n = 13 (8 patients; 5 healthy control); Error bar indicates standard error mean. The student's t-test was performed independently for each gene between healthy peripheral blood samples and patients’ peripheral blood samples. *p* = non-significant

In an effort to use the identified mRNAs as biomarkers for the detection of the atherosclerotic condition, we quantified the levels of a few mRNAs in patients’ blood by qRT-PCR and compared them with that of healthy blood samples. The panel included the mRNAs that showed altered expression in both the plaques and specifically in ES or AS plaques (Table 2). The qRT-PCR result showed up-regulation of genes that were involved in the chemokine signaling pathway, cytokine-cytokine receptor interaction, and complement and coagulation cascades while the expression of genes that were functioning in the TGF-beta signaling pathway was found to be lowered as compared to healthy controls (Fig. 5). We also performed QRT-PCR for the miRNA-mRNA pairs found in our study (Table 1). An Expression pattern similar to the microarray analysis was found. However, the mRNAs and miRNAs showed a non-significant *p-*value. Increasing the sample size may improve the significance of our result. These results support the presence of differentially expressed miRNA and mRNA in patients’ blood and their use as potential biomarkers for assessing the stage and risk of atherosclerosis.

**Table 2:** The panel of genes proposed in the study to be used as biomarker for diagnosis of plaque stage

	ES	AS
*Chemokine signalling pathway and cytokine-cytokine receptor interaction*		
SPP1	↑^*^	**↑↑**
CCL18	**↑↑**	**↑**
CXCL9	**↑**	**↑↑**
CXCL12	**↑**	**↑↑**
IL13RA1	**↑**	**↓**^†^
TNFSF14	**↓**	**↑**
CXCR4	**↓**	**↑**
CCL19	**↑**	
TNF	**↑**	
IL7R		**↑**
IL2RG		**↑**
ARRB2		**↑**
IL10RA		**↑**
*Complement and coagulation cascades*		
C1QC	**↑**	**↑↑**
C1QB	**↑**	**↑↑**
F13A1		**↑**
CFH		**↑**
SERPINE1		**↑**
*TGF-beta signaling pathway*		
BMPR2		**↓**
TGFB3		**↓**
TGFBR2		**↓**

*Up-regulated, ^†^down-regulated

## Discussion

Atherosclerosis is a chronic inflammatory condition of the arteries. Various effector immune mechanisms and signaling networks are involved in the pathology of atherosclerotic plaque formation [39–44]. Elevated levels of inflammatory cytokines and chemokines have been observed in various studies on affected cardiac tissues [45–47]. However, studies on differential expression of immune/ inflammatory genes in plaques of early versus advanced stages remain scant. Volger et al, 2007, studied gene expression profiles of endothelium from human large arteries having focal atherosclerosis of the ES or AS [32]. The transcriptomes of the lesional arteries were compared with the transcriptomes of their unaffected sides, thus limiting genetic and external confounders. The authors, however, collected samples post-mortem after the disease. Post-mortem tissues undergo significant changes in their physical and chemical compositions possibly altering the profile of mRNA stability retrieval and amplification required for transcriptome profiling [48]. Therefore, we performed a microarray analysis of ES and AS plaque as well as healthy LIMA tissue obtained surgically from the same live individuals undergoing CABG Surgery.

**Fig. 6:**
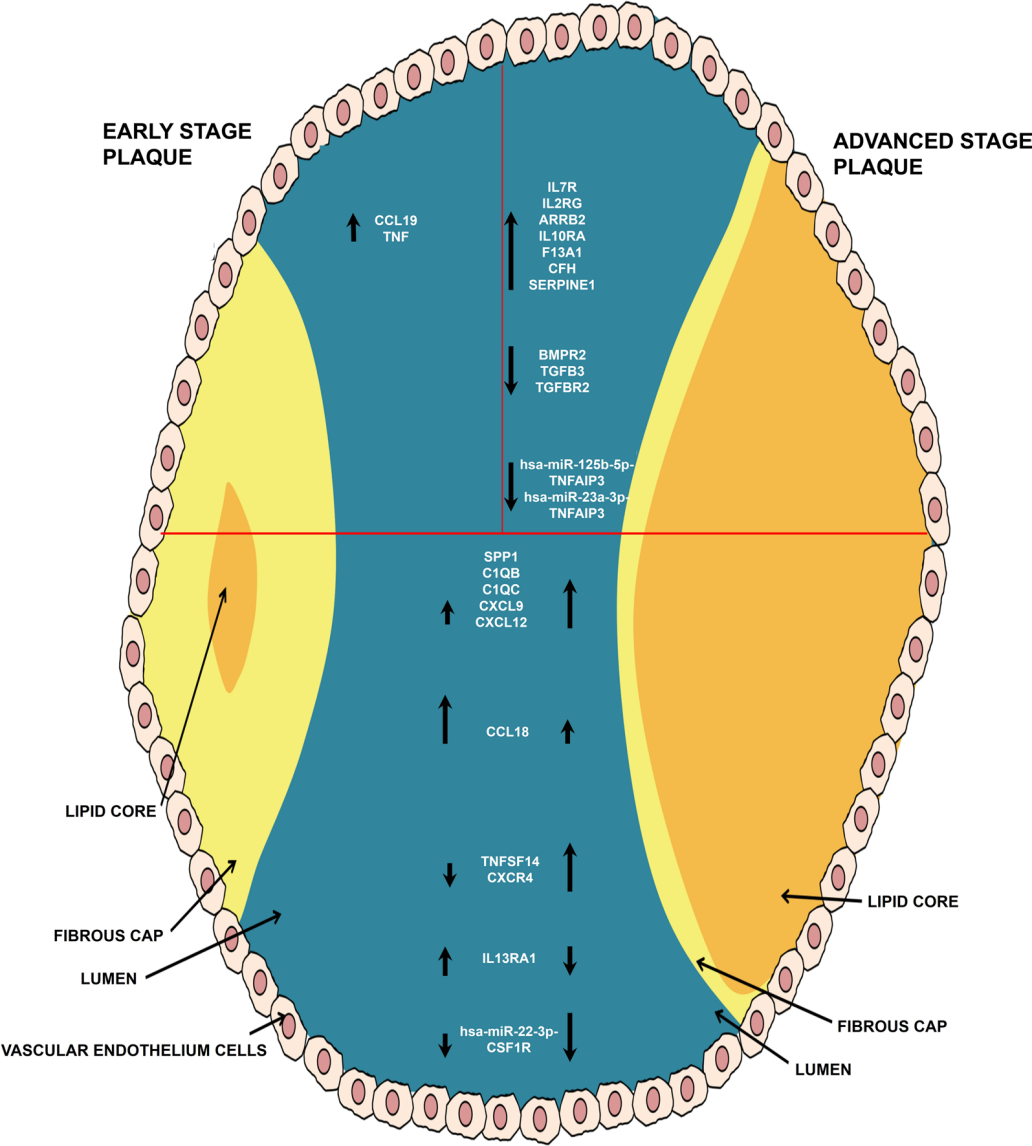
Summary of mRNA and miRNA expression analysis in atherosclerotic plaques as compared to LIMA. The upper left panel represents the differentially expressed in early stage plaques only. The upper right panel represents the differentially expressed mRNA and miRNA along with miRNA-mRNA pairs in advanced stage plaques only. The lower panel shows mRNA, miRNA, and miRNA-mRNA pairs differentially expressed in both the plaques

Here, we attempted to understand the differential expression profiles of immune response-related genes in early versus advanced atherosclerotic plaques. We found that the ES and AS plaques are associated with differential expression of various mRNAs and miRNAs, suggesting that the transcriptional regulations and responses in the initial stage of plaque formation may contribute to the subsequent pathophysiology of atherosclerosis (Fig. 6). In both the plaques, significant numbers of genes were found to be involved in more than one arm of the immune system. Interestingly, few of these genes have already been reported to be involved in atherosclerosis pathology, supporting the validity of our data. Examples of such genes are SPP1, CXCL9, CCL18, FcγRIIIA, etc. [23–28]. The higher expression patterns of common genes in AS versus ES plaques suggest that atherosclerosis involves an array of processes where alterations in their profiles shift the balance from ES to AS stage evolution. A prominent example of such a common gene is SPP1, required for macrophage chemotaxis, critical in lipid peroxidation, and a pathological landmark of atherosclerosis [49, 50]. Our results also unravel the differentially expressed genes in plaque in stage specific manner, and definitive profiles marking the dominance of early and/or advanced atherosclerotic changes in patients. For example, the chemokine CCL19 was up-regulated only in ES plaque, which might be involved in the trafficking of various inflammatory cells at the plaque site. On the other hand, AS plaques showed up-regulation of genes involved in stronger inflammation (e.g.- C1QC, C1QB, TYROBP, etc.).

The over-expression of genes functioning in the chemokine signaling pathway in ES plaques suggests the initiation of various immune cells recruitment at the affected tissue. For example, CCL18 for naïve T cells, CCL19 for T and B cell migration, and CCL5 for blood monocytes, memory T helper cells, and eosinophils [51–53]. The genes (like CCL5, TNF, CXCL10) which are involved in cytokine-cytokine signaling stimulate these incoming immune cells to release various other signaling and pro-inflammatory molecules; for instance, activation of eosinophils by CCL5 and stimulation of monocytes by CXCL10 [53]. Therefore, chemotaxis and activation of a diverse immune cell population in ES plaque suggest an onset of inflammation in the affected vascular tissue. The up-regulation of genes coding for different cytokine receptors like IL7R, IL2RG, IFNGR2, and IL17RA only in AS plaque and the absence of expression of their respective cytokines in microarray analysis indicates a compensatory phenomenon in which cells attempt to enhance the probability of binding to the scarce cytokines. Also, the suppression of the genes required for TGF-beta signaling in AS plaque and ES plaque suggests a failure of immune-regulatory mechanisms to check the on-going inflammation.

In an attempt to understand the regulation of differentially expressed mRNAs at the post-transcriptional level, microarray analysis against miRNAs for both plaques was carried out, followed by the identification of miRNA-mRNA interaction pairs for the corresponding plaques. Few of the resulting miRNAs like hsa-miR-17, hsa-miR-16, hsa-miR-195, hsa-miR-27a, hsa-miR-20b, etc. have already been reported in other studies to be associated with coronary artery disease [54–56]. We found three miRNA-mRNA pairs associated with atherosclerosis. Among them, one pair included hsa-miR-125b and its target TNFAIP3. Hsa-miR-125b act as a negative regulator of inflammatory genes and TNFAIP3 is involved in the cytokine-mediated immune and inflammatory responses [57]. Therefore, the involvement of hsa-miR-125b in inflammation during atherosclerosis can also be proposed via TNFAIP3. The second interaction pair included hsa-miR-22-3p and its target CSF1R. Since CSF1R promotes the release of pro-inflammatory chemokines, the finding that this gene is up-regulated while the miRNA targeting it is down-regulated in ES and AS plaques suggest the role of this miRNA-mRNA pair in atherosclerosis associated inflammation.

Identifying microarray-based biomarkers may complement and help in circumventing the invasive techniques for clinical evaluation of  the stage of atherosclerotic pathophysiology and its implication in patient prognosis and healthcare. Circulating mRNAs and miRNAs are being investigated as possible biomarkers for the early detection and progression of various diseases [58–60]. Moreno et al. (2009) reported that the plasma level of CD163-TWEAK (tumor necrosis factor-like weak inducer of apoptosis) is a potential biomarker of atherosclerosis [61]. Therefore, differentially expressed mRNAs and miRNAs profiles identified in the present study may reveal a fingerprint of the presence and/or the dominance of ES and AS plaques. We correlated the same in the peripheral blood of the patients intending to identify the significant changes in their blood. The pattern of expression difference was similar to that reported in microarray analysis, supporting the hypothesis that the expression levels of common and stage specific mRNA and miRNAs in the blood samples of patients can be used as a biomarker for early detection and monitoring of progression and/or regression of coronary atherosclerosis and thus possibly to evaluate the effectiveness of anti-atherosclerotic therapies on a larger scale. However, we need a proper scoring system for the expression of these stage specific genes so that weightage to ES or AS could be given. Although we cannot know the frequency of the particular plaque stage in the patient, the ratio of the expression of stage specific genes can tell us the condition of more prevalent plaque.

## Conclusions

From our results, two distinct mRNA-miRNA pair profiles are evident:

- Panel 1−SPP1**↑**, CCL18**↑↑**, CXCL9**↑**, CXCL12**↑**, IL13AR1**↑**, TNFSF14**↓**, CXCR4**↓**, CCL19**↑**, TNF**↑**, C1QC**↑**, C1QB**↑**
- Panel 2–SPP1**↑↑**, CCL18**↑**, CXCL9**↑↑**, CXCL12**↑↑**, IL13AR1**↓**, TNFSF14**↑**, CXCR4**↑**, IL7R**↑**, IL2RG**↑**, ARRB2**↑**, IL10RA**↑,** C1QC**↑↑**, C1QB**↑↑**

The former has a high correlation with ES plaque and later with AS plaque. Such profiles are also reflected in the peripheral blood. Therefore, we propose that ratios of these profiles may signature the proportion of advanced pathology in patients. This might supplement the imaging analysis of atherosclerotic plaques and enable clinicians to better assess the disease state and plan for clinical management accordingly.

## Supplementary Information

**Additional file 1.** The list of primers used for qRT-PCR.

**Additional file 2.** Significantly-enriched inflammation associated KEGG pathways of differentially expressed genes in early stage and advanced stage atherosclerotic plaque.

**Additional file 3.** Differentially expressed mRNAs.

**Additional file 4.** Differentially expressed miRNAs.

## Data Availability

The datasets generated and/or analyzed during the current study are available in the European Bioinformatics Institute Array Express repository, (accession number-E-MTAB-10052 (mRNA data); E-MTAB-10051 (miRNA data)).

## Abbreviations

CVD: Cardiovascular diseases
IHD: Ischemic heart disease
CAD: Coronary artery disease
ES: Early stage
AS: Advanced stage
LIMA: Left internal mammary artery
CABG: Coronary artery bypass grafting
KEGG: Kyoto encyclopaedia of genes and genomes
