# Supplementary material for: Signature transcriptome analysis of stage specific atherosclerotic plaques of patients
Source: BMC Med Genomics. 2022 Apr 29;15:99. doi: 10.1186/s12920-022-01250-8 (PMC9055692; doi:10.1186/s12920-022-01250-8)
Supplement: Supplementary file 1 — Additional file 1. The list of primers used for qRT-PCR. [file 12920_2022_1250_MOESM1_ESM.docx]

*Additional File 1: The list of primers used for qRT-PCR.*

| **GENE** | **FORWARD PRIMER** | **REVERSE PRIMER** |
| --- | --- | --- |
| TNF | CTCTTCTGCCTGCTGCACTTTG | ATGGGCTACAGGCTTGTCACTC |
| SPP1 | CGAGGTGATAGTGTGGTTTATGG | GCACCATTCAACTCCTCGCTTTC |
| CCL18 | GTTGACTATTCTGAAACCAGCCC | GTCGCTGATGTATTTCTGGACCC |
| CXCL9 | CTGTTCCTGCATCAGCACCAAC | TGAACTCCATTCTTCAGTGTAGCA |
| IL7R | ATCGCAGCACTCACTGACCTGT | TCAGGCACTTTACCTCCACGAG |
| CXCL12 | CTCAACACTCCAAACTGTGCCC | CTCCAGGTACTCCTGAATCCAC |
| IL13RA1 | CCTGAATGAGAGGATTTGTCTGC | CAGTCACAGCAGACTCAGGATC |
| TNFSF14 | GGTCTCTTGCTGTTGCTGATGG | TTGACCTCGTGAGACCTTCGCT |
| CXCR4 | CTCCTCTTTGTCATCACGCTTCC | GGATGAGGACACTGCTGTAGAG |
| CCL19 | CGTGAGGAACTTCCACTACCTTC | GTCTCTGGATGATGCGTTCTACC |
| IL2RG | CACTCTGTGGAAGTGCTCAGCA | GAGCCAACAGAGATAACCACGG |
| ARRB2 | CTGACTACCTGAAGGACCGCAA | GTGGCGATGAACAGGTCTTTGC |
| IL10RA | GCCGAAAGAAGCTACCCAGTGT | GGTCCAAGTTCTTCAGCTCTGG |
| C1QC | CAACACAGGCTGCTACGGGATC | CTGCCCTTTGGGTCCTCGGAT |
| C1QB | CGGGTATCCCTGGGACACCTG | GCCGACTTTTCCTGGATTCCCA |
| F13A1 | CAACAGCCACAACCGTTACACC | CTTGGATCAGCACCGCCTCTTT |
| CFH | ATTCCGTTCAGTGCTACCACTTTG | CCATTGAGGAGTTCAGGAGGTG |
| SERPINE1 | CTCATCAGCCACTGGAAAGGCA | GACTCGTGAAGTCAGCCTGAAAC |
| BMPR2 | AGAGACCCAAGTTCCCAGAAGC | CCTTTCCTCAGCACACTGTGCA |
| TGFB3 | CTAAGCGGAATGAGCAGAGGATC | TCTCAACAGCCACTCACGCACA |
| TGFBR2 | GTCTGTGGATGACCTGGCTAAC | GACATCGGTCTGCTTGAAGGAC |
| GAPDH | GTCTCCTCTGACTTCAACAGCG | ACCACCCTGTTGCTGTAGCCAA |
| TNFAIP3 | CTCAACTGGTGTCGAGAAGTCC | TTCCTTGAGCGTGCTGAACAGC |
| CSF1R | GCTGCCTTACAACGAGAAGTGG | CATCCTCCTTGCCCAGACCAAA |
| hsa-miR-125b-5p | TCCCTGAGACCCTAACTTGTGA |  |
| hsa-mir-23a-3p | ATCACATTGCCAGGGATTTCC |  |
| hsa-miR-22-3p | AAGCTGCCAGTTGAAGAACTGT |  |
