# Supplementary material for: Signature transcriptome analysis of stage specific atherosclerotic plaques of patients
Source: BMC Med Genomics. 2022 Apr 29;15:99. doi: 10.1186/s12920-022-01250-8 (PMC9055692; doi:10.1186/s12920-022-01250-8)
Supplement: Supplementary file 3 — Additional file 3. Differentially expressed mRNAs. [file 12920_2022_1250_MOESM3_ESM.docx]

*Additional File 2: Significantly-enriched inflammation associated KEGG pathways of differentially expressed genes in early stage and advanced stage atherosclerotic plaque.*

|  | PATHWAY | GENES (FOLD CHANGE) | | | | | | | | | |
| --- | --- | --- | --- | --- | --- | --- | --- | --- | --- | --- | --- |
| ES  (UP-REGULATED) | Chemokine signaling pathway | CCL18 (33.08) | CCL19 (2.81) | CCL5 (2.65) | CCR2  (2.01) | CXCL10 (4.7) | CXCL12 (3.37) | CXCL16 (2.04) | CXCL9 (3.73) | GRK4  (2.24) | PIK3CG (2.04) |
|  |  | PREX1 (2.37) | SHC4 (2.25) |  |  |  |  |  |  |  |  |
|  | Cytokine-cytokine receptor interaction | CCL18 (33.08) | CCL19 (2.81) | CCL5 (2.65) | CCR2  (2.01) | CSF1R (2.49) | CXCL10 (4.7) | CXCL12 (3.37) | CXCL16 (2.04) | CXCL9 (3.73) | EDA2R (2.36) |
|  |  | IL13RA1 (2.03) | TGFB1 (2.34) | TNF (2.52) | TNFRSF11B (3.68) | SPP1 (680.14) |  |  |  |  |  |
| AS  (UP-REGULATED) | Cytokine-cytokine receptor interaction | CCL18 (16.06) | CCL5 (6.2) | CCR1 (2.6) | CD27  (2.3) | CSF1R (-2.34) | CX3CR1 (4.62) | CXCL10 (4.05) | CXCL11 (2.13) | CXCL12 (8.51) | CXCL16 (3.11) |
|  |  | CXCL9 (29.97) | CXCR4 (8.56) | EDA2R (4.08) | IFNA10 (2.1) | IFNAR2 (2.8) | IFNGR2 (2.03) | IL10RA (4.83) | IL17RA (2.52) | IL18 (2.43) | IL23A (2.12) |
|  |  | IL2RA (2.41) | IL2RG (8.15) | IL7R (10.03) | TGFB1 (5.57) | TNFRSF11B (4.78) | TNFSF10 (3.5) | TNFSF13B (2.19) | TNFSF14 (2.1) |  |  |
|  | Chemokine signaling pathway | ARRB2 (5.06) | CCL18 (16.06) | CCL5 (6.2) | CCR1  (2.6) | CX3CR1 (4.62) | CXCL10 (4.05) | CXCL11 (2.13) | CXCL12 (8.51) | CXCL16 (3.11) | CXCL9 (29.97) |
|  |  | CXCR4 (8.56) | DOCK2 (3.5) | HCK (2.06) | LYN  (3.11) | PIK3CG (3.8) | PLCB2 (2.72) | RAC2 (2.51) | STAT1 (3.23) | SPP1 (2955.37) |  |
|  | Complement and coagulation cascades | C1QB (5.68) | C1QC (27.84) | C2  (2.4) | C3AR1 (4.22) | C4A  (3.71) | C4B (2.65) | CFH  (4.81) | CR1 (2.74) | F13A1 (5.31) | SERPINE1 (3.98) |
|  |  | VWF (3.49) |  |  |  |  |  |  |  |  |  |
| AS  (DOWN-REGULATED) | TGF-beta signaling pathway | BMPR1A (-3.66) | BMPR1B (-2.41) | BMPR2 (-2.42) | CREBBP  (-2.34) | DCN  (-6.6) | ID2  (-2.97) | ID4  (-6.48) | LTBP1  (-7.02) | MYC (3.17) | RHOA  (-3.29) |
|  |  | ROCK1 (-2.75) | SMAD9 (-2.32) | SMURF2  (-2.52) | TGFB3  (-2.87) | TGFBR2  (-2.05) |  |  |  |  |  |
